# Magnesium promotes vascularization and osseointegration in diabetic states

**DOI:** 10.1038/s41368-023-00271-y

**Published:** 2024-01-31

**Authors:** Linfeng Liu, Feiyu Wang, Wei Song, Danting Zhang, Weimin Lin, Qi Yin, Qian Wang, Hanwen Li, Quan Yuan, Shiwen Zhang

**Affiliations:** 1 State Key Laboratory of Oral Diseases & National Center for Stomatology & National Clinical Research Center for Oral Diseases & West China Hospital of Stomatology, Sichuan University, Chengdu, China; 2 State Key Laboratory of Oral Diseases & National Center for Stomatology & National Clinical Research Center for Oral Diseases & Department of Oral Implantology, West China Hospital of Stomatology, Sichuan University, Chengdu, China

**Keywords:** Implants, Biomaterials - cells

## Abstract

Diabetes has long been considered a risk factor in implant therapy and impaired wound healing in soft and hard oral tissues. Magnesium has been proved to promote bone healing under normal conditions. Here, we elucidate the mechanism by which Mg^2+^ promotes angiogenesis and osseointegration in diabetic status. We generated a diabetic mice model and demonstrated the alveolar bone healing was compromised, with significantly decreased angiogenesis. We then developed Mg-coating implants with hydrothermal synthesis. These implants successfully improved the vascularization and osseointegration in diabetic status. Mechanically, Mg^2+^ promoted the degradation of Kelch-like ECH-associated protein 1 (Keap1) and the nucleation of nuclear factor erythroid 2-related factor 2 (Nrf2) by up-regulating the expression of sestrin 2 (SESN2) in endothelial cells, thus reducing the elevated levels of oxidative stress in mitochondria and relieving endothelial cell dysfunction under hyperglycemia. Altogether, our data suggested that Mg^2+^ promoted angiogenesis and osseointegration in diabetic mice by regulating endothelial mitochondrial metabolism.

## Introduction

As the preferred option for restoring missing teeth, dental implants are becoming widely popularized in clinical practice.^1^ However, implant failure still occurs due to pathological or physiological reasons such as inflammation, systemic diseases, aging, and hormone levels.^2^ Among the factors, diabetes has long been considered a risk factor in implant therapy and impaired wound healing in soft and hard oral tissues,^3–6^ for which effective solutions are still lacking. Unfortunately, according to the research of the International Diabetes Federation (IDF) in 2021, about 573 million adults (10.5%) have diabetes in the world. Substantial evidence suggests that diabetes negatively interferes with the tissue healing process of bone healing, resulting in poor bone regeneration and delayed healing time.^7^ Developing corresponding treatment strategies, such as innovation in implant materials, is still a challenge.

Neovascularization plays a critical role in implant osseointegration,^8^ which transports hormones, oxygen, and nutrients for bone formation and remodeling in the local microenvironment, providing an ecological niche for skeletal stem cells from proliferation to osteogenic differentiation.^9^ Nevertheless, vascular damage is instrumental in many diabetic pathological changes. Diabetes along with associated vascular ailments and endothelial cell dysfunction leads to delayed wound healing by inhibiting angiogenesis due to hyperinflammatory states and metabolic disorders of endothelial cells.^10,11^ Recently, massive studies have demonstrated to accelerate diabetic wound healing by promoting angiogenesis in diabetic states.^12^

As one of the indispensable mineral elements of the human body, magnesium plays a critical role in maintaining normal metabolism^13^ and extensive studies have confirmed that Mg^2+^ promotes osteogenesis.^14–16^ Interestingly, recent studies have proved that Mg^2+^ improves endothelial cell function and reduces the endothelial cell oxidative stress levels,^2,17,18^ but the mechanism of Mg^2+^ regulating endothelial cells under hyperglycemia is still lacking. In addition, a growing body of literature has focused on the use of oral magnesium supplements to improve glucose metabolism in people with diabetes or those at risk of diabetes, suggesting that magnesium deficiency is strongly associated with the progression of diabetes.^19–21^

Here, we sought to explore the effect of Mg-coating implants on vascularization and osseointegration in diabetic mice by screening Mg^2+^ concentration in vitro and constructing Mg-coating implants. Biological processes at the implant tissue interface are critical for implant osseointegration. However, it is difficult to comprehensively and three-dimensionally present the implant bonding interface and the surrounding micro-environment through traditional histological techniques.^22,23^ To solve the problem we adopted the PEGASOS tissue-clearing method.^24,25^ By immersing samples into various chemical substances, the implant‐bone interface becomes transparent with internal structures intact and fluorescence preserved. Combining the PEGASOS tissue-clearing method with fluorescently labeled transgenic mice, we could trace angiogenesis and osteogenesis processes at the implant-bone interface in diabetic states.

## Results

### Diabetes mellitus (DM) impairs vascularization and bone regeneration in alveolar bone healing

**Fig. 1:**
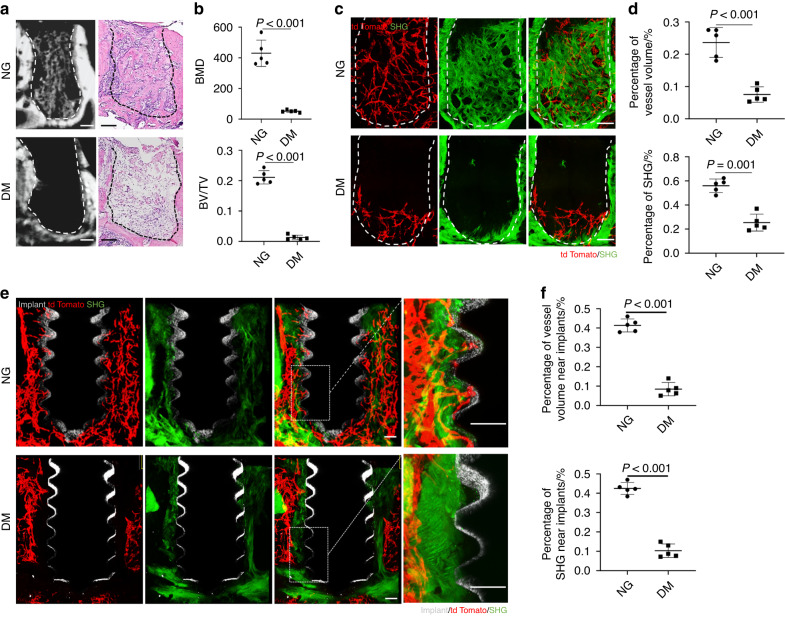
Diabetes mellitus (DM) impairs vascularization and bone regeneration in alveolar bone healing. **a** Representative images of μCT reconstruction and H&E staining of the sockets 7 days post tooth extraction in NG and DM mice. Scale bar = 100 μm. **b** Quantitative μCT analyses of newly formed bone in the sockets (*n* = 5). **c** Tissue clearing-based 3-dimensional images of extraction sockets on day 7 after surgery with tdTomato signal displaying blood vessels (red) and second harmonic generation (SHG) signal displaying bone (green). Scale bar = 100 μm. **d** Quantitative analyses of vessel volume and SHG in three-dimensional images of the sockets. **e** Tissue clearing-based three-dimensional images of *Cdh5-Cre*^*ERT2*^*;tdTomato* mice showed angiogenesis and osteogenesis around implants in NG and DM mice on day 7 after surgery. Scale bar = 100 μm. **f** Quantitative analyses of vessel volume and SHG around implants

To detect the effect of diabetes on the healing of alveolar bone, we generated a diabetic mice model by STZ injection and extracted the first mandibular molars. Micro-CT analysis showed that DM mice had a significant reduction in bone formation compared to normoglycemic (NG) mice. H&E staining of the decalcified alveolar bone confirmed the empty extraction socket histomorphology in DM mice in contrast to obvious bone formation in NG mice (Fig. 1a, b).

Next, we sought to investigate the impact of diabetes mellitus on angiogenesis in the alveolar bone healing. We generated *Cdh5-Cre*^*ERT2*^*;tdTomato* transgenic mice to label endothelial cells. Samples were treated with PEGASOS tissue clearing method and scanned by a multiphoton microscope. We observed that the extraction sockets were filled with Tomato^+^ endothelial cells in NG mice. These cells were connected to form a 3D network and traveled through newly generated bone collagen, which was in green by second harmonic signals (SHG) (Fig. 1c). However, in diabetic mice, Tomato^+^ cells were only scattered at the bottom of the socket (Fig. 1c). The proportion of blood vessels in the socket was significantly reduced and the collagen signals was almost absent (Fig. 1c, d).

Similarly, through deep imaging of the peri-implant environment, we found that there were significant Tomato^+^ cells aggregation, and new bone formation around the implant screw in NG mice, while Tomato^+^ cells and green collagen signals were also largely reduced in diabetic mice (Fig. 1e, f).

### Mg^2+^ ameliorates endothelial cell dysfunction under hyperglycemia

**Fig. 2:**
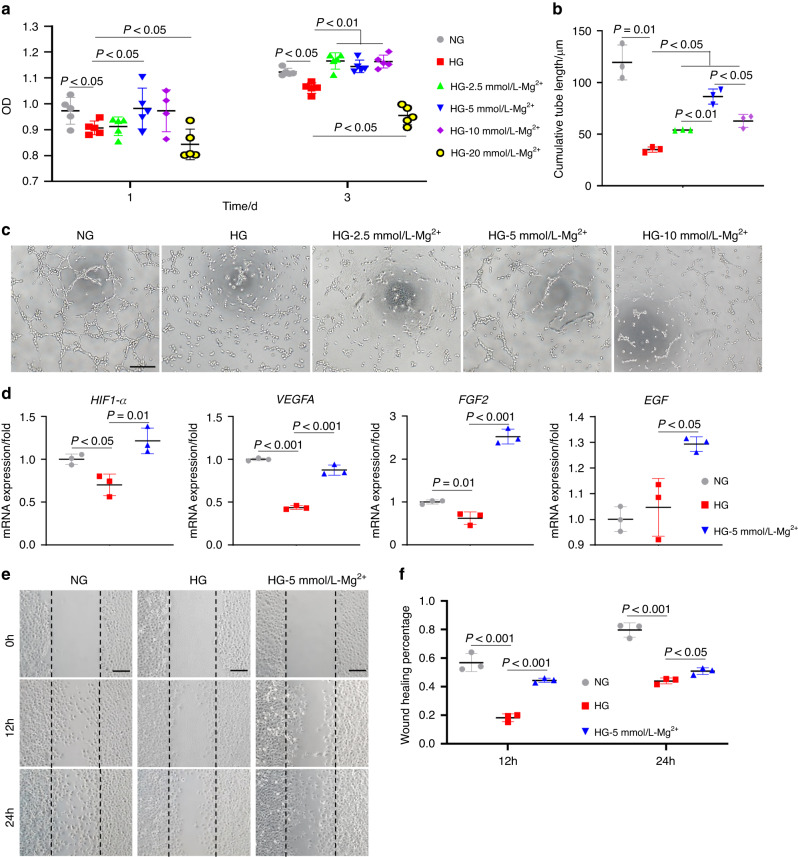
Mg^2+^ ameliorates endothelial cell dysfunction under hyperglycemia. **a** Cell proliferation assay of HUVECs by CCK-8 test (*n* = 5). **b** Quantitative analyses of tube length (*n* = 3). **c** Representative images in tube formation assay showed the angiogenesis in vitro. Scale bar = 200 μm. **d** qRT-PCR analyses of the expression of *HIF1-α, VEGFA, FGF2, EGF* (*n* = 3). **e** Representative images of the migration of HUVECs in a wound-healing assay. Scale bar = 200 μm. **f** Quantitative analyses of cell migration with the wound healing percentage (*n* = 3)

Next, we sought to investigate the effect of Mg^2+^ on human umbilical vein endothelial cells (HUVECs) under hyperglycemia. The cell counting kit 8 (CCK-8) assay showed that the proliferation of endothelial cells was significantly inhibited on day 1 and day 3 in the high glucose (HG) medium. On day 1, endothelial cells exposed to HG medium containing 5 mmol/L Mg^2+^ showed an obvious ability to promote endothelial cell proliferation. Instead, when the Mg^2+^ concentration reached 20 mM, cell proliferation was inhibited. This phenomenon was even more pronounced on day 3 (*P* < 0.05) (Fig. 2a).

To further explore the optimal Mg^2+^ concentration for the endothelial angiogenesis under hyperglycemia, a tube formation assay was performed to evaluate angiogenesis in vitro. Compared to the normal glucose (NG) group, the HG group showed apparent less tube formation (*P* = 0.01). Though 2.5 mmol/L, 5 mmol/L, and 10 mmol/L all had facilitated tube formation under hyperglycemia (*P* < 0.05), the 5 mmol/L-HG group exhibited upper promotion compared to the other two groups (Fig. 2b, c). qPCR results indicated that 5 mmol/L Mg^2+^ could partially rescue the expression of angiogenesis-related genes, such as *HIF1-α*, *VEGFA*, *EGF*, and *FGF2* that were suppressed in the HG group (Fig. 2d). In addition, we adopted wound-healing experiments and observed that 5 mmol/L Mg^2+^ accelerated endothelial cell migration under hyperglycemia (Fig. 2e, f).

### Synthesis and characterization of Mg-coating implants

**Fig. 3:**
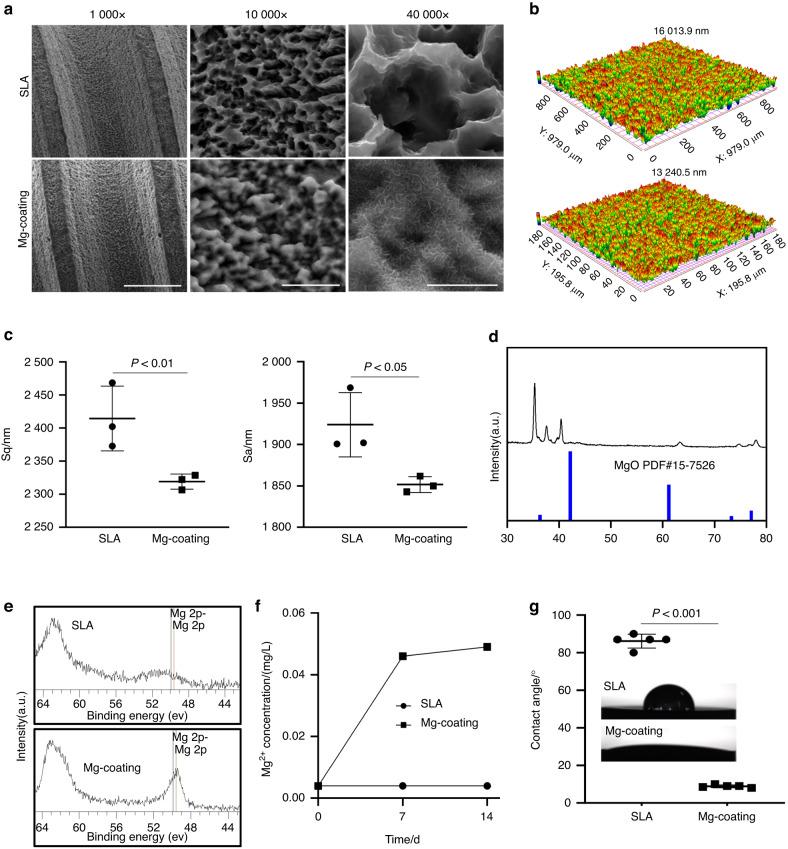
Synthesis and characterization of Mg-coating implants. **a** SEM scanning images of the implants. Scale = 100 μm, 10 μm, 5 μm in turn. **b**, **c** Surface topography of the samples. **d**, **e** XPS and XRD showed the elemental composition of the samples. **f** The release of Mg^2+^. **g** Contact angle and representative images of droplets on different samples (*n* = 5)

Next, we developed Mg-coating implants and sheets by hydrothermal synthesis. SEM scanning showed that the coating retained threaded construction structure of the implant while homogeneous inorganic salts were deposited on the implant surface in a nanopattern-like structure, but did not block the pores of the SLA implant surface under higher magnification (Fig. 3a). The surface roughness of the material was slightly reduced from the surface contour reconstruction and measurement (Fig. 3b, c). The coating structure was further validated by X-ray diffraction (XRD) in which typical characteristic peaks of MgO (PDF#15-7526) at 2θ were detected (Fig. 3d). Hence, the modality of Mg-coatings, MgO, was corroborated. XPS confirmed the successful embellishment of Mg elements on the implants (Fig. 3e). Ion release experiments exhibited the liberation from the coatings in simulated body fluids which peaked within 7 days (Fig. 3f). While the water contact angle (WCA) reduced from 89° to 9° in the Mg-coating group (Fig. 3g), and the improvement of hydrophilicity has been proved to promote cell adhesion and spread.

**Fig. 4:**
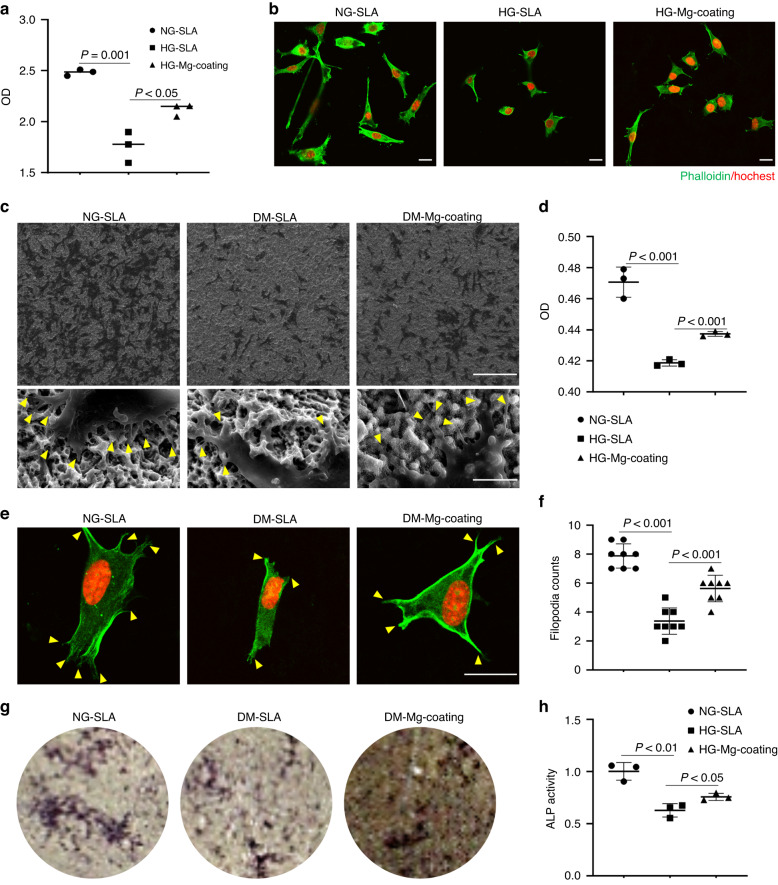
Biological testing of implants. **a** Cell proliferation assay of HUVECs cultured on Mg-coating samples by CCK-8 test (*n* = 3). **b** Representative images of the morphology of HUVECs on different samples under NG or HG. Scale bar = 50 μm. **c** SEM morphology of MC3T3-E1 osteoblasts on titanium and Mg-coating titanium surface under NG or HG. Scale bar = 250 μm (upper), 10 μm (lower). **d** Cell proliferation assay of MC3T3-E1 osteoblasts by CCK-8 (*n* = 3). **e**, **f** Representative images morphology and filopodia counts of MC3T3-E1 osteoblasts on titanium and Mg-coating titanium surface under NG or HG. Scale bar = 50 μm. **g** ALP staining. **h** ALP activity

Mg-coating group exhibited better endothelial cell proliferation under hyperglycemia (Fig. 4a). Similarly, by hoechst/phalloidin cytoskeleton staining, we observed fewer cells in SLA group while the Mg-coating group partly rescued this phenomenon under hyperglycemia (Fig. 4b). In addition to endothelial cell-mediated angiogenesis, osteoblastic precursor cells recruiting around the implant and differentiating to osteoblasts also played a crucial role in osseointegration. Experimental consequences evidenced Mg-coating group possessed more excellent pseudopodia protrusion, cell proliferation, and osteogenic differentiation of MC3T3-E1 osteoblasts (Fig. 4c–g).

### Mg^2+^ promotes vascularization and osseointegration in diabetic mice

**Fig. 5:**
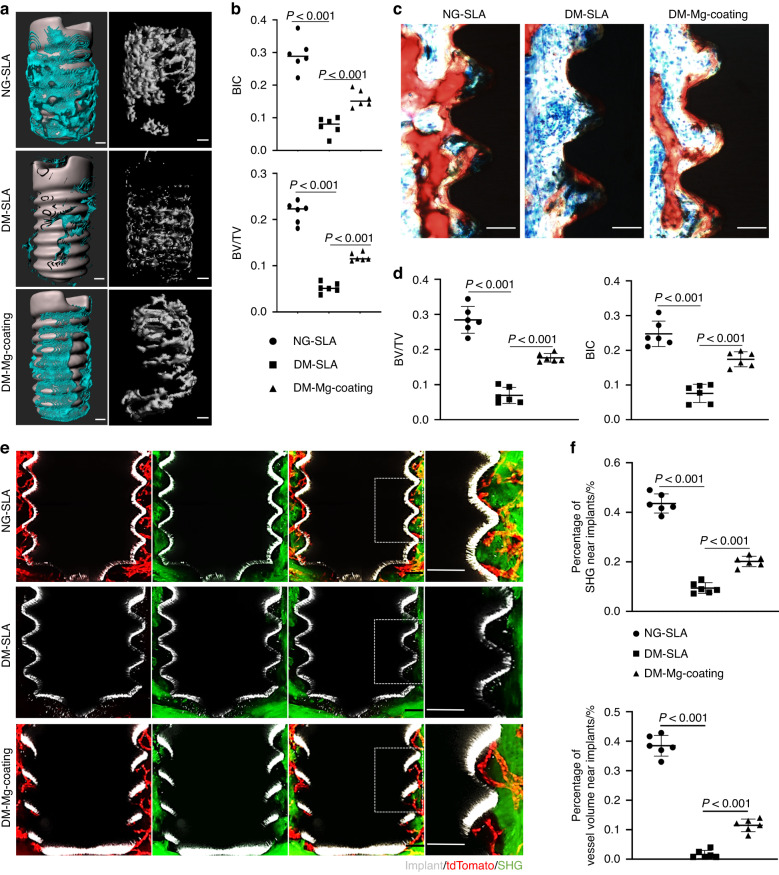
Mg^2+^ promotes vascularization and osseointegration in diabetic mice. **a** Representative images of μCT reconstruction of the newly formed bone (blue) with implants and without implants (gray) 7 days post-surgery. Scale bar = 100 μm. **b** Quantitative analyses of bone-implant contact rate (BIC) and bone volume/tissue volume (BV/TV) (*n* = 6). **c** Representative images of the histological sections, red for bone. Scale bar = 50 μm. **d** Quantitative analyses of BIC and BV/TV in histological sections (*n* = 6). **e** Tissue clearing–based three-dimensional images around implants showed angiogenesis and osteogenesis on day 7 after surgery, with tdTomato signal displaying blood vessels (red), second harmonic generation (SHG) signal displaying bone (green) and implant (gray). Scale bar = 100 μm. **f** Quantitative analyses of vessel volume and SHG around implants (*n* = 6)

To verify the effect of Mg-coating implants on vascularization and osseointegration in diabetic mice, we inserted implants in *Cdh-Cre*^*ERT2*^*;tdTomato* mice. Visually, CT analysis revealed the Mg-coating implants exhibited higher bone-implant contact rate (BIC) and bone volume/tissue volume (BV/TV) in DM mice (Fig. 5a, b). Histomorphometric analyses further corroborated that more new bone tissue was filled in the neighboring area to the surface of the implants in Mg-coating group (Fig. 5c, d). In turn, more Tomato^+^ cells were recruited around the Mg-coating implants in fluorescently labeled mice. These increased cells indicated enriched angiogenesis in DM mice. While more bone collagen in green shown by the SHG signal was visualized around the Mg-coating implants in diabetic states (Fig. 5e, f). Altogether, through in vivo model validation, we demonstrated the ability of Mg-coating implants to promote inhibited vascularization and osseointegration in diabetic states.

However, in previous inspections, we found changes in the surface topography and hydrophilicity of the material. We constructed titanium-coating implants and proved the surface topography, roughness, and wettability were no different from magnesium-coating implants (Fig.S2a–e). Furthermore, in vivo experiments proved that Mg-coating implants still promoted peri-implant osseointegration under diabetes compared to titanium-coatings implants, confirming the osteogenic effect of Mg^2+^ in diabetic states independent of roughness and wettability (Fig. S2f, g).

### Mg^2+^ reduces endothelial cell oxidative stress levels under hyperglycemia

**Fig. 6:**
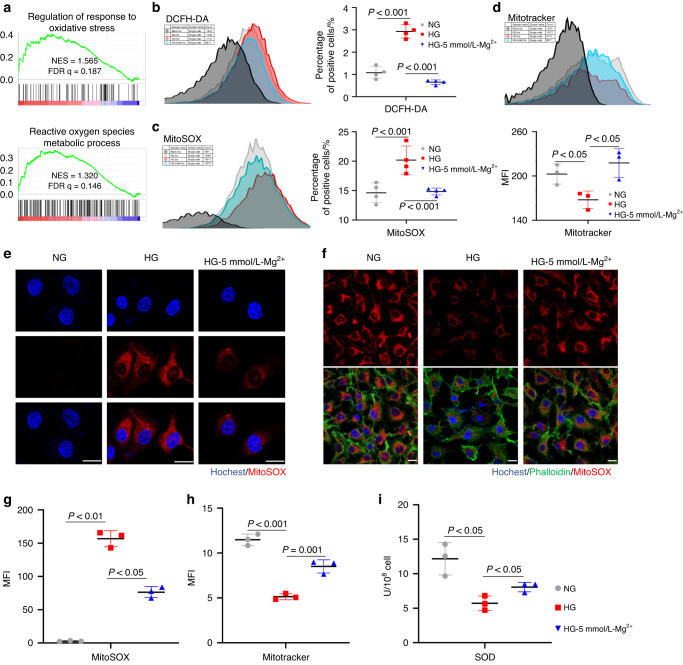
Mg^2+^ reduces endothelial cell oxidative stress levels under hyperglycemia. **a** Gene set enrichment analysis (GSEA) for response to oxidative stress. **b** HUVECs stained with DCFH-DA was analyzed by flow cytometry (*n* = 3). **c** HUVECs stained with MitoSOX was analyzed by flow cytometry (*n* = 3). **d** HUVECs stained with Mitotracker was analyzed by flow cytometry (*n* = 3). **e** Representative fluorescence images of MitoSOX staining for mitochondrial ROS. Scale bar = 50 μm (*n* = 3). **f** Representative fluorescence images and quantification of Mitotracker staining. Scale bar = 50 μm (*n* = 3). **g** Immunofluorescence intensity quantification of MitoSOX staining (*n* = 3). **h** Immunofluorescence intensity quantification of Mitotracker staining. (*n* = 3). **I** SOD activity detection

RNA-sequencing analysis was performed to explore the mechanism by which Mg^2+^ improved endothelial cell function under hyperglycemia. GESA analysis suggested the regulation of response to oxidative stress and the reactive oxygen species metabolic process were upregulated in the Mg^2+^ group (Fig. 6a). 2′,7′-dichlorodihydrofluorescein diacetate (DCFH-DA) flow cytometry showed that the level of oxidative stress in endothelial cells in the HG group was significantly increased, while this increase recovered after the addition of Mg^2+^ (Fig. 6b). Intracellular reactive oxygen species (ROS) were mainly produced by mitochondria. Further detection of mitochondrial ROS indicated the purging ability of Mg^2+^ on mitochondrial ROS accumulation by flow cytometry and immunofluorescent staining (Fig. 6c, e, g). High reactive oxygen species result in metabolic disorders and even death of cells by mitochondrial attack. Mitochondrial quality measuring proved that Mg^2+^ rescued the damaged mitochondrial mass under hyperglycemia (Fig. 6d, f, h). Superoxide dismutase (SOD), a crucial antioxidant metalloenzyme modulated by nucleated Nrf2, manifested enhanced activity in the Mg^2+^ group under hyperglycemia (*P* < 0.05) (Fig. 6i).

**Fig. 7:**
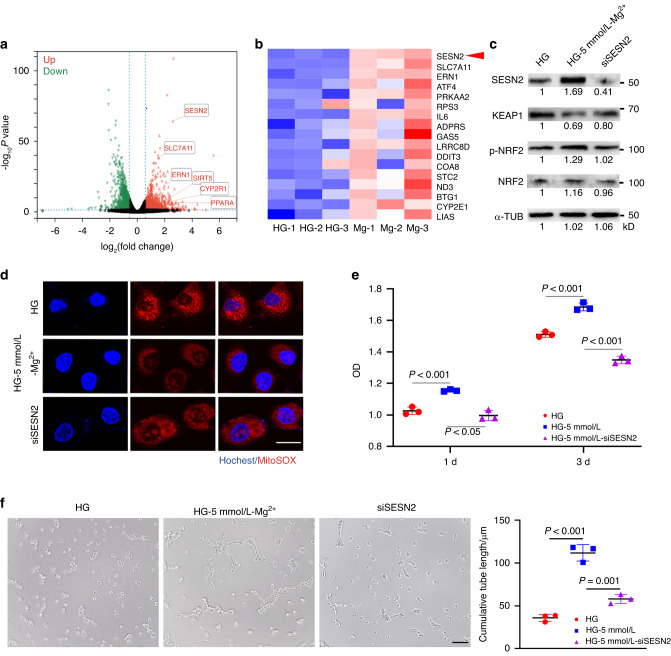
Mg^2+^ upregulates SESN2 in HUVECs under hyperglycemia. **a** Volcano of differentially expressed genes. **b** Heatmap of representative genes involved in response to oxidative stress. **c** Western blots of SESN2, KEAP1, nucleated NRF2 and total NRF2 in HUVECs. **d** Representative fluorescence images of MitoSOX staining for mitochondrial ROS. Scale bar = 50 μm (*n* = 3). **e** Cell proliferation assay of HUVECs by CCK-8 test (*n* = 3). **F** Representative images and quantitative analyses in tube formation assay showed the angiogenesis in vitro. Scale bar = 200 μm (*n* = 3)

Digging deeper into the mechanism, we found that SESN2 was significantly upregulated in RNA sequencing (Fig. 7a, b) which has been proved to promote Keap1 degradation and activate Nrf2 nucleation to protect cells from oxidative stress.

Then, we depleted SESN2 in HUVECs using small interfering RNAs (siRNA) and western blot confirmed the decline of Keap1 and the upregulation of nucleated Nrf2 in the Mg^2+^ group were reversed after depleting SESN2 (Fig. 7c). Immunofluorescent staining also proved mitochondrial ROS accumulated (Fig. 7d) and cell functions including proliferation and angiogenesis in vitro were also impaired in 5 mmol/L-HG group after depleting SESN2 (Fig. 7e, f).

## Discussion

Post-traumatic revascularization is a critical step in bone healing, providing oxygen, nutrients, and ions necessary for stem cells to differentiate into osteoblasts and matrix mineralization for damage repair.^26,27^ In the process of bone development and repair, angiogenesis is closely related to osteogenesis.^28,29^ However, diabetes leads to various vascular complications and inhibits angiogenesis, resulting in poor wound healing.^30,31^ Our data also suggested that diabetes seriously impaired vascularization and bone regeneration in alveolar bone healing, which strongly supported the work presenting delayed healing and increased alveolar destruction from Devlin et al.^32^. In recent years, numerous studies have been conducted to promote the healing of diabetic soft and hard tissues by promoting angiogenesis.^10,18^

Mg^2+^ has been widely recognized in promoting osteogenesis.^14^ Recently, Huang et al. (2021) created a multifunctional magnesium organic framework-based microneedle patch for accelerating diabetic wound healing through accelerating angiogenesis.^18^ Liu et al. (2023) also constructed magnesium-containing hydrogels to strengthen diabetic wound angiogenesis to promote healing.^33^ However, at present, whether Mg^2+^ could promote peri-implant vascularization and osseointegration under diabetes and its intrinsic mechanism are still unknown. In our study, Mg^2+^ was demonstrated for the first time to improve peri-implant vascularization and osseointegration in diabetic mice.

Endothelial cells (ECs), on the other hand, are thought to be the driving force behind angiogenesis,^34^ quickly initiating the formation of new blood vessels during wound healing.^35^ In diabetes, impaired endothelial cell function leads to disturbed angiogenesis, and our in vitro experiments demonstrated that Mg^2+^ could improve endothelial cell proliferation, tube formation, and migration under hyperglycemia.

Hyperglycemia-induced endothelial cell disorders were proved to be mainly related to increased levels of intracellular oxidative stress and excess intracellular superoxide,^36,37^ resulting in dysfunction. While mitochondria were an important source of intracellular ROS,^37,38^ increased intracellular mitochondrial proton gradient in a hyperglycemic state inhibits electron transfer from reduced coenzyme Q (ubiquinone) to electron transport chain complex III. Instead, electrons are transferred to molecular oxygen, leading to the production of superoxide.^39^ Excess ROS destroys cellular proteins, lipids, mitochondria, and DNA, leading to fatal damage to cells.^34,37^

Our RNA sequencing reflected that the addition of Mg^2+^ increased the reactive oxygen species metabolic process of endothelial cells, while the detection of ROS in endothelial cells supported the ability of Mg^2+^ to reduce the level of mitochondrial oxidative stress elevated under hyperglycemia.

Through further exploration of the sequencing results, we found that the expression of SESN2 and ATF4 in the Mg^2+^ group increased significantly. ATF4 is a key gene in mitochondrial stress regulation and is involved in gene transcription for antioxidant responses, autophagy, amino acid biosynthesis, and transport. Studies have shown that the upregulation of ATF4 could mitigate mitochondrial ROS production and ROS-mediated mitochondrial damage.^40^ SESN2 is a stress-induced protein that can increase the nuclear translocation of Nrf2 and improve the transcriptional activity mediated by Nrf2/antioxidant response element (ARE), thereby exerting antioxidant capacity.^41^ Nrf2 is an important transcription factor in regulating cellular oxidative stress and a central regulator for maintaining redox homeostasis in cells.^42^ Under normal circumstances, Nrf2 will be modified by Keap1 complex ubiquitination and degradation in the cytoplasm, after Nrf2 is transferred to the nucleus, it will form a complex with coactivators and bind to promoter regions (AREs), thereby inducing and regulating the compositional and inducible expression of a series of antioxidant proteins, which can reduce cell damage caused by reactive oxygen species and electrophiles, keep cells in a stable state, and maintain redox homeostasis.^42^

Using small interfering RNAs (siRNA) we eliminated SESN2 in HUVECs. The upregulation of Keap1 and the decline of nucleated Nrf2 confirmed the decline of Keap1 after eliminating SESN2 in the Mg^2+^ group strongly demonstrated that Mg^2+^ regulates the degradation of Keap1 and the nucleation of Nrf2 through upregulating SESN2. Further detection of elevated mitochondrial ROS and impaired cell proliferation and angiogenesis in vitro proved Mg^2+^ mediated antioxidant response and repaired endothelial cell dysfunction through SESN2. However, absence of SESN2 did not completely inhibit the effect of Mg^2+^ on endothelial cells under hyperglycemia, suggesting other regulatory mechanisms such as ATF4 or SLC7A11 might make an impact. In the sequencing data that the expression of Solute Carrier Family 7 Member 11 (SLC7A11), one of the most critical upstream regulators of ferrozogenesis, was also significantly upregulated. Combined with our experimental data that Mg^2+^ promoted the survival of high-glucose endothelial cells, reduced cellular oxidative stress levels, improved mitochondrial quality, increased Nrf2 nucleation upstream of SLC7A11 and upregulate ATF4 levels, we speculated that Mg^2+^ might inhibit ferrozogenesis in high-glucose endothelial cells, which also requires further verification.

At the same time, the implant-tissue interface is a key issue in implant research. Due to the limitations of traditional technical means, it was difficult to visually observe the changes in the implant-osseointegration interface in three dimensions. Drawing on the tissue clearing technology previously developed by our research group,^25^ we visually observed the changes in the peri-implant three-dimensional microenvironment of diabetic mice for the first time by preserving endogenous fluorescence expression. Our data profoundly proved that diabetes seriously impaired the vascularization and bone regeneration in alveolar bone healing while it was also the first comprehensively and three-dimensionally display in which angiogenesis was inhibited in diabetic tooth extraction socket and implant osseointegration. Of course, in the construction of the material we also changed the surface roughness and wettability, which had been reported to have an effect on angiogenesis and bone formation.^43^ In order to exclude the effects of roughness and wettability, we used titanium dioxide as the coating to achieve roughness and hydrophilicity similar to Mg-coating. In vivo experiments, we found that the increase of wettability could slightly increase bone formation, but the Mg-coating still showed a significant effect of promoting peri-implant osseointegration. Combined with our cytology experiments, Mg^2+^ were added to the medium to confirm the effect of magnesium on endothelial cells. We further confirmed that Mg^2+^ played a crucial role in promoting vascularization and osteogenesis, independent of other factors. However, in this study we focused on Mg^2+^ promoting vascularization and osteogenesis in diabetic states, therefore control group of normoglycemic animals with Mg-coating implants was absent. We also lacked the experiments to knock out SESN2 or NRF2 in mice to further support our results, which needed further exploration.

In summary, we demonstrated that Mg^2+^ promoted Nrf2 nucleation through SESN2 upregulation, thereby reducing the level of mitochondrial oxidative stress in endothelial cells under hyperglycemia in vitro and promoting peri-implant vascularization and osseointegration in diabetic mice.

## Materials and methods

### Mice

The study conforms to the ARRIVE Guidelines. All protocols for animal care and experiments were reviewed and approved by the Subcommittee on Research and Animal Care of Sichuan University. All mice were C57BL/6 background and housed in a specific pathogen-free environment with a 12 h light-dark cycle. Rosa26-tdTomato (007905) was from Jackson Laboratory. *Cdh5-Cre*^*ERT2*^ mice were kindly provided by Prof. Bi-Sen Ding’s lab (State Key Laboratory of Biotherapy, Sichuan University). *Cdh5-Cre*^*ERT2*^*;tdTomato* mice were generated by mating *Cdh5-Cre*^*ERT2*^ mice with *Rosa26-tdTomato* mice. 1.5 mg per 10 g tamoxifen was injected intraperitoneally daily for 2 days consecutively to light up fluorescence before surgery.

### Construction of the diabetic model

STZ (Shanghai Maokang Biotechnology Co., Ltd.) was intraperitoneally 40 mg/kg injected into mice consecutively for 5 days to induce a model. With serum glucose level reaching 16.67 mmol/L for 2 weeks, surgery was performed (Fig. S1).

### Surgery

First mandibular molars were removed under anesthesia with a 26 G syringe needle and forceps. For immediate implant placement, a self-designed sandblasted, large grit and acid-etched (SLA) titanium implant (0.6-mm diameter; WEGO, China) was screwed in the socket after extraction under the stereomicroscope. On day 7 after extraction or implantation, mandibles were harvested.

### PEGASOS tissue clearing process

The PEGASOS tissue clearing process was performed as reported before.^25^

### Image acquisition and analyses

Images were acquired through a multiphoton microscope (Lecia SP8 DIVE). TdTomato signals were acquired under a 561 nm excitation laser line. The implant was acquired with a sapphire laser at 950 nm wavelength. Second harmonic signals (SHG) imaging was acquired with a non‐descanned detector. IMARIS software (version 9.1.2; Oxford Instruments) was used for data analysis and image processing. A region 50 μm near the thread grooves of each implant was used for analyses. For each sample, three randomly selected regions were used for quantification.

### Micro-computed tomography images and analyses

The harvested mandibles with implants were fixed in 4% PFA at 4 °C for 24 h and then stored in 0.5% PFA at 4 °C before being scanned. The samples were scanned with a μCT-50 system (Scanco Medical) at a medium resolution and a voxel size of 7 μm. IMARIS software (version 9.1.2; Oxford Instruments) was used for data analysis and image processing.

### Histologic preparation and staining

The mandibles with implants were fixed in 4% PFA at 4 °C for 1 day, followed by 4 weeks of decalcification in 10% EDTA (pH 7.4) at 4 °C with shaking. Mandibles for hematoxylin and eosin (H&E) staining were dehydrated in graded ethanol and embedded in paraffin. The paraffin blocks were cut by a microtome (Leica RM2255) into 5 μm-thick sections. H&E staining was performed according to the manufacturer’s instructions (Biosharp). A region 50 μm near the thread grooves of each implant was used for analyses. For each sample, three randomly selected regions were used for quantification.

### Cell culture

Human Umbilical Vein Endothelial Cells (HUVECs) provided by State Key Laboratory of Biotherapy, West China Second University Hospital, Sichuan University, were cultured in Dulbecco’s modified Eagle medium (DMEM) (Hyclone, Laboratories, Logan, UT, USA) containing 10% fetal bovine serum (Gibco, Grand Island, NY, USA), 100 U/mL penicillin (Hyclone, Cytiva, Marlborough, MA, USA) and 100 μg/mL streptomycin (Hyclone, Cytiva, Marlborough, MA, USA). The culture media were changed every other day. HUVEC were cultured in three different conditions: normal glucose (NG, 5.5 mmol/L), high glucose (HG, 35 mmol/L), and HG+Mg^2+^ (MgSO_4_, MACKLIN, China).

MC3T3-E1 osteoblasts were cultured in alpha-modified Eagle medium (α-MEM) (Hyclone, Laboratories, Logan, UT, USA) and changed every other day.

### Cell counting kit-8 assay

HUVECs were seeded in a 96-well plate at a density of 5 000 cells per well. Then NG medium, HG medium, or HG medium containing 2.5, 5, 10, or 20 mmol/L Mg^2+^ was added. Cell counting kit-8 (CCK-8) assay was conducted after 24 h and 72 h separately. Optical density (OD) values were measured by a spectrophotometer (Thermo Fisher Scientific, Waltham, MA, USA) at 450 nm.

### Tube formation test

100 μL growth-factor-reduced Matrigel (BD Biosciences, USA) was added into each well of the 96-well plate and then place in a 37 °C incubator for 30 min until solidification. Next, HUVECs (5 000 cells per well) were seeded on the solidified Matrigel substrates and cultured in serum-free NG medium, HG medium, or HG medium containing 2.5, 5, and 10 mmol/L Mg^2+^. After 6 h of incubation (5% CO_2_, 37 °C), the cells were imaged using an inverted fluorescence microscope (Olympus, Japan). The tube length was measured using ImageJ software (NIH, USA).

### Quantitative reverse-transcription PCR (qRT-PCR)

HUVECs were cultured in NG medium, HG medium, or HG medium containing 5 mmol/L Mg^2+^ for 24 h. Then total RNA of HUVECs was extracted by Trizol Reagent (Invitrogen, Carlsbad, CA, USA). Reverse transcription complementary DNA (cDNA) from 1 μL of RNA using the PrimeScript RT Kit (TaKaRa Bio, Otsu, Japan), and the genes of interest were measured by SYBR Premix Ex Taq II (TaKaRa) in LightCycler 96 (Roche, Basel, Switzerland). Relative microRNA (mRNA) expression was analyzed using a delta delta comparative threshold cycle (2^-ΔΔCt^) method by normalizing with glyceraldehyde-3-phosphate dehydrogenase (GADPH).

### Scratch wound healing assay

HUVECs were seeded in a 6-well plate and cultured till 100% confluence, and the 10% FBS medium was replaced with 1% FBS medium for starvation culture 24 h after scratches. A 200 μL pipette tip was used to trace along the center of the hole along the ruler, leaving even and straight scratches. Subsequently, the medium was replaced with NG medium, HG medium, or HG medium containing 5 mmol/L Mg^2+^. The wound closure was observed and pictured after 12 h and 24 h. ImageJ software was used to calculate the proportion of the healing area.

### Gene knockdown

We diluted 2 μg siRNA duplex into 250 μL DMEM as solution A and diluted 5 μL Lipofectamine RNAiMAX (Invitrogen, USA) into 250 μL DMEM for solution B. A and B were mixed and incubated 30 min at RT. When the cells reached 40 to 70% confluence, 500 μL mixture was added to 2 mL of normal growth medium without antibiotics for 12 h at 37°C.

### Fabrication of Mg-coating and Ti-coating implants

For hydrothermal synthesis, an aqueous solution of MgO (or TiO_2_) and NaOH was prepared in ddwater. Mix the reactants completely and the solutions were combined and subsequently stirred at room temperature for 5 min. The final stock solution was transferred to a hydrothermal reactor and the processed titanium was put in the reactor at the same time. The hydrothermal autoclave was sealed and set up to 180°C for 12 h. Then the plates were washed, respectively, by ddwater and ethanol twice for 10 min. The plates were placed in an oven and heated at 70°C for 1 h for drying.

### Materials characterization of samples

The surface topography of the samples was scanned by a field-emission SEM (FE-SEM; JSM-7500 F, JEOL, Japan). Elemental analysis was performed by EDS. Superview W1 (China) were used for detect surface topography. XRD (LabX, Shimadzu, Japan) was used to scan the structures of the samples. The elemental composition and chemical state of surfaces were measured by XPS (AXIS Supra, Kratos, USA). The water contact angle was performed by the WCA apparatus (Zhongchen Digital Technic Apparatus Co., Shanghai, China). The inductively coupled plasma atomic emission spectroscopy (ICP-AES, 5100 SVDV, USA) was used to assess the release of Mg^2+^ in PBS.

### Cell viability assay

HUVECs or MC3T3-E1 osteoblasts (1 × 10^4^ cells per well) were seeded onto NG + SLA, HG + SLA, and HG+Mg-coating plates in the 48-well plates for 24 h. Cell counting kit-8 (CCK-8) assay was conducted as before.

### Cell morphology and immunofluorescence staining

Phalloidin (Shanghai Maokang Biotechnology Co., Ltd.) was used to observe the cytoskeleton. After 24 h of incubation, cells were fixed with 4% PFA for 10 min and the cell cytoskeleton was stained by phalloidin and the nuclei stained by DAPI. Fluorescent imaging of cells was detected by laser scanning confocal microscopy (FV3000; Olympus, Japan). After 1 day of culture on substrates, cells were fixed overnight with 2.5% glutaraldehyde. Then, the samples were dehydrated sequentially in gradient ethanol (30%, 50%, 75%, 85%, 95%, 100%) for 15 min for SEM observation.

### Alkaline phosphatase staining

MC3T3-E1 osteoblasts were cultured with the osteogenic medium on substrates for 7 days and fixed with 4% PFA for 20 min. Then staining was performed with a commercial Alkaline Phosphatase Assay Kit (Beyotime Biotechnology, Shanghai, China). ALP activity quantitation was performed with a commercial kit (Beyotime Biotechnology, Shanghai, China) and detected by a spectrophotometer (Thermo Fisher Scientific, USA) at 450 nm.

### Histologic and histomorphometric analyses

7 days after implant surgery, mandibles containing implants were collected and fixed in 10% buffered formalin at 4°C for 1 week. Samples were dehydrated by increasing concentrations of ethanol (60%, 80%, 90%, 100%) and embedded in light-curing epoxy resin (TechnoVit7200VLC; Hereaus-Kulzer, Wehrheim, Germany). The embedded specimens were cut parallel to the long axis of the implant. Then, the specimens were ground to around 80 μm thickness using a grinding machine (ExaktApparatebau, Norderstedt, Germany). Sections were stained with Stevenel’s blue and Van Gieson’s stain and then observed under a light microscope (Olympus, Japan).

### RNA-Sequencing and data analysis

HUVECs were treated with HG medium or HG medium containing 5 mmol/L Mg^2+^ for 48 h (*n* = 3 in each group). Total RNA was extracted with Trizol reagent (Invitrogen, Carlsbad, CA, USA) and purified with poly-T oligo-attached magnetic beads. FastQC (S-Andrews, v0.11.5) was used to guarantee the quality of RNA-Sequencing (RNA-Seq) data and by using HISAT2 (DaehwanKimLab,v.2.0.5) we mapped them to Mus musculus reference genomes. Genes were considered significantly differentially expressed if showing fold change ≥ 1 and *P*-value ≤ 0.05. For Gene Set Enrichment Analysis (GSEA), the gene lists were downloaded from the GSEA database (www.gsea-msigdb.org/gsea/). GSEA was processed with GSEA software (https://www.gsea-msigdb.org/gsea/index.jsp).

### Flow cytometry analysis

HUVECs were treated with NG medium, HG medium, or HG medium containing 5 mmol/L Mg^2+^ for 48 h. 2′,7′-dichlorodihydrofluorescein diacetate (DCFH-DA), and MitoSOX staining were performed following a modified protocol. The samples were then tested by flow cytometry (BD Biosciences, USA), and the flow cytometry data were analyzed using Flowjo software.

### Western blot

HUVECs were treated with HG medium or HG medium containing 5 mmol/L Mg^2+^ for 48 h. Total proteins were extracted by protein extraction kit (Signalway Antibody, Greenbelt, MD, USA). Proteins concentrations were measured with BCA Protein Assay Kit (Pierce, Rockford, IL, USA;23225). Protein was separated equally in 8% SDS–polyacrylamide gel electrophoresis (SDS-PAGE) gels and transferred to 0.22 μm polyvinylidene fluoride (PVDF) membranes (Thermo Fisher Scientific, Waltham, MA, USA). After blocking in 5% skim milk, membranes were immersed with primary antibodies and corresponding secondary antibodies. Proteins were developed by a gel imaging system (Bio-Rad Laboratories, Hercules, CA, USA) with Immobilon Reagents (Millipore, Billerica, MA, USA).

### SOD activity assay

SOD activity assay was performed as directed (Beyotime, China).

### Statistical analyses

All data were expressed as mean ± standard deviation (SD). For each group, at least three independent experiments were conducted with duplicate samples. Statistical analysis was done with a one-way ANOVA followed by Bonferroni’s multiple comparison test. *P* ≤ 0.05 was considered statistically significant.

## Data and materials availability

The RNA sequencing data set has been deposited into the NCBI database with the identifier GSE236139.

### Supplementary information

supplemental material
