# Supplementary material for: Magnesium promotes vascularization and osseointegration in diabetic states
Source: Int J Oral Sci. 2024 Jan 31;16:10. doi: 10.1038/s41368-023-00271-y (PMC10831079; doi:10.1038/s41368-023-00271-y)
Supplement: Supplementary file 1 — supplemental material [file 41368_2023_271_MOESM1_ESM.docx]

Supplementary file for

**Magnesium promotes vascularization and osseointegration in diabetic states**

Linfeng Liu^1,#^, Feiyu Wang^1,#^ Wei Song^1^, Danting Zhang^1^, Weimin Lin^1^, Qi Yin^1^, Qian Wang^1^, Hanwen Li^1^, Quan Yuan^1,2^*, Shiwen Zhang^1，2^*

*1. State Key Laboratory of Oral Diseases & National Center for Stomatology & National Clinical Research Center for Oral Diseases, West China Hospital of Stomatology, Sichuan University, Chengdu 610041, Sichuan, China*

*2.Department of Oral Implantology, West China Hospital of Stomatology, Sichuan University, Chengdu, China*

**Corresponding Authors:*

S. Zhang, State Key Laboratory of Oral Diseases, West China Hospital of Stomatology, Sichuan University

#14 Third Section, Renmin Road South, Chengdu, 610041, China.

Email: [sw.zhang2018@scu.edu.cn](mailto:sw.zhang2018@scu.edu.cn)

Q. Yuan, State Key Laboratory of Oral Diseases, West China Hospital of Stomatology, Sichuan University

#14 Third Section, Renmin Road South, Chengdu, 610041, China.

Email: [yuanquan@scu.edu.cn](mailto:yuanquan@scu.edu.cn)

^#^ Linfeng Liu and Feiyu Wang contributed equally to this work.

**This file includes:**

Supplementary Figures and Legends


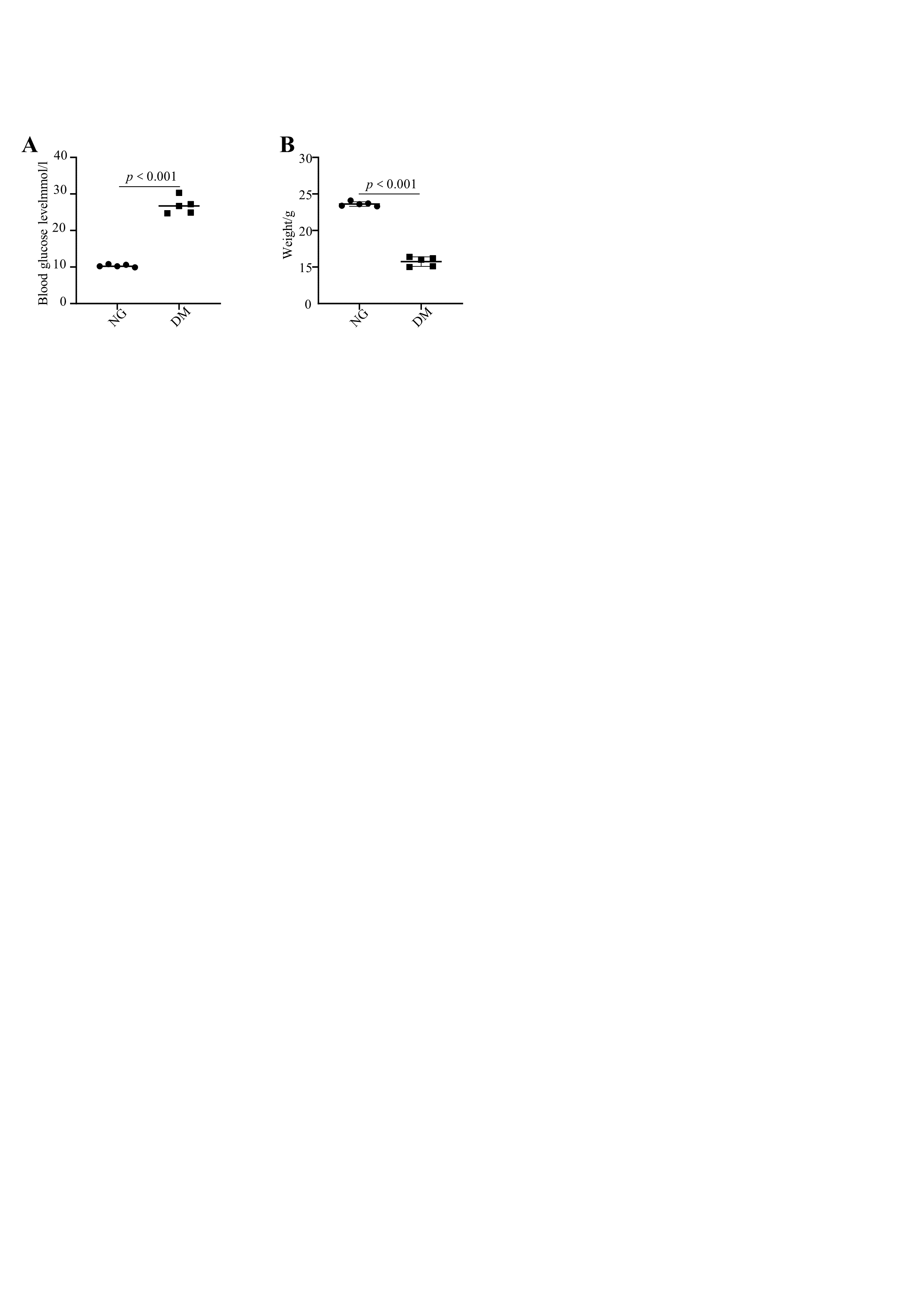


**Supplementary Figure 1 Blood glucose level and body weight of mice.**

1. Blood glucose level of mice. (B) Body weight of mice.


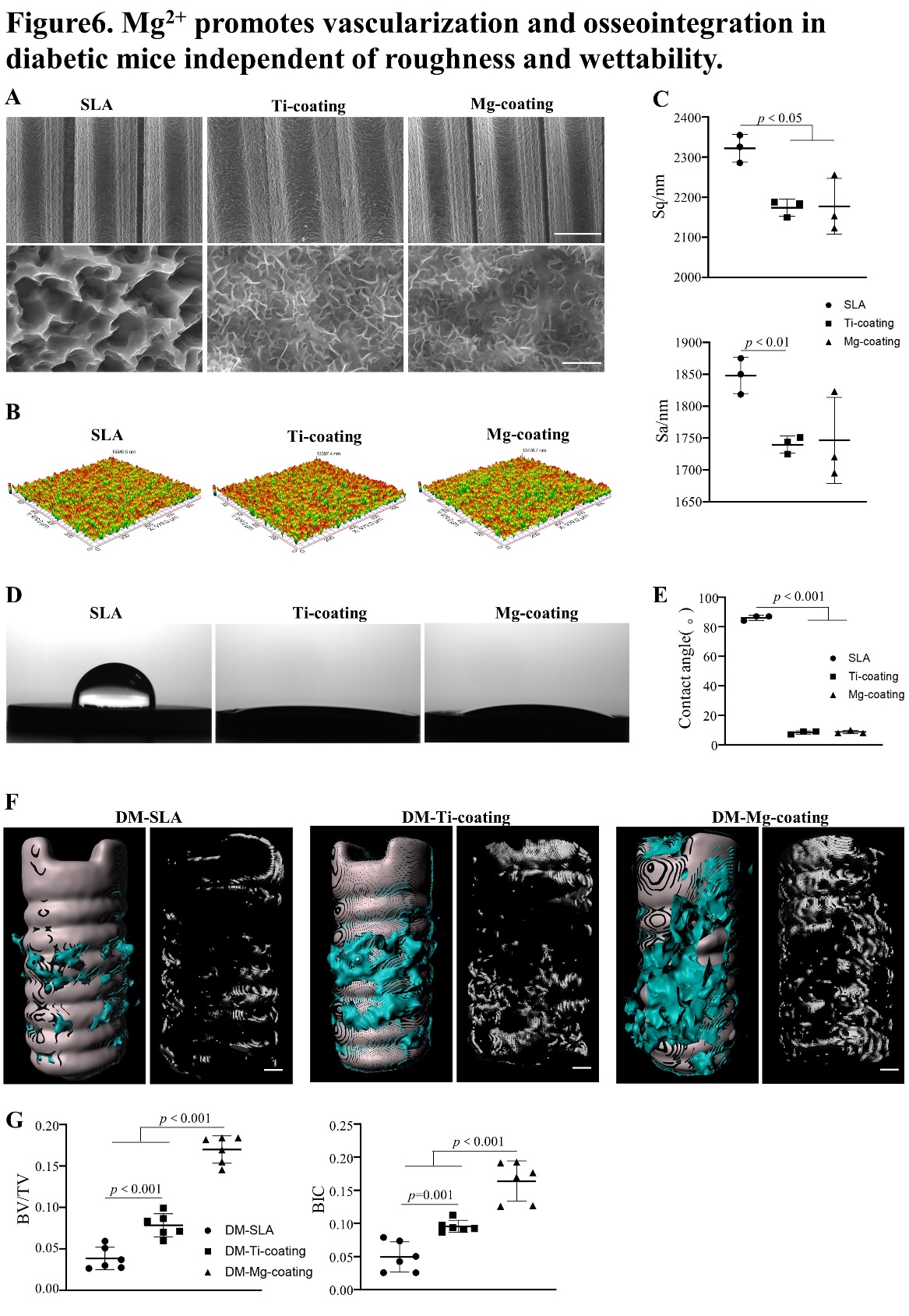


**Supplementary Figure 2 Mg^2+^ promotes vascularization and osseointegration in diabetic mice independent of roughness and wettability.**

(A) SEM scanning images of the Ti-coating and Mg-coating implants. (B-C) Surface topography of the Ti-coating and Mg-coating implants. (D-E) Contact angle and representative images of droplets on different samples. (F) Representative images of μCT reconstruction of the newly formed bone (blue) with implants and without implants (gray) 7 days post-surgery. Scale bar = 100 μm. (G) Quantitative analyses of bone-implant contact rate (BIC) and bone volume/tissue volume (BV/TV) (n = 6).
